# Supplementary material for: Solar wind erosion of lunar regolith is suppressed by surface morphology and regolith properties
Source: Commun Earth Environ. 2025 Jul 16;6(1):560. doi: 10.1038/s43247-025-02546-0 (PMC12267057; doi:10.1038/s43247-025-02546-0)
Supplement: Supplementary file 2 — Supplementary Information [file 43247_2025_2546_MOESM2_ESM.pdf]

Supplementary Information to
Solar wind erosion of lunar regolith is suppressed by surface morphology and regolith properties

Johannes Brötzner,[1,*] Herbert Biber,[1] Paul Stefan Szabo,[2] Noah Jäggi,[3] Lea Fuchs,[1] Andreas Nenning,[4] Martina Fellinger,[1] Gyula Nagy,[1] Eduardo Pitthan,[5] Daniel Primetzhofer,[5] Andreas Mutzke,[6] Richard Arthur Wilhelm,[1] Peter Wurz,[7] André Galli,[7] and Friedrich Aumayr[1]

[1] *Institute of Applied Physics, TU Wien, Wiedner Hauptstraße 8-10/E134, A-1040 Vienna, Austria*
[2] *Space Sciences Laboratory, University of California, 7 Gauss Way, Berkeley, 94720 CA, USA*
[3] *Material Science and Engineering Department, University of Virginia, 395 McCormick Road, Charlottesville, VA 22904, USA*
[4] *Institute of Chemical Technologies and Analytics, TU Wien, Getreidemarkt 9, A-1060 Vienna, Austria*
[5] *Department of Physics and Astronomy, Uppsala University, Box 516, SE-752 37 Uppsala, Sweden*
[6] *Max Planck Institute for Plasma Physics, Wendelsteinstraße 1, DE-17491 Greifswald, Germany*
[7] *Space Science and Planetology, Physics Institute, University of Bern, Sidlerstrasse 5, CH-3012 Bern, Switzerland*

(Dated: June 25, 2025)

Supplementary Discussion

SPRAY Simulations and Morphology Effects

SPRAY is a code developed by Cupak *et al.* [1] in order to extend one-dimensional BCA simulations to three-dimensional sample surfaces without the need to calculate the full collision cascade. At the core of its working principle, it relies on an input of 1D BCA simulations for the desired target-projectile combination, as well as input on surfaces of interest. For a perfectly flat surface, SPRAY reproduces the 1D input and can thus never be more accurate than the underlying simulations. The surface input comes from microscopy images, typically AFM, and therefore represents not any rough surface, but physically available samples.

The input surface is triangulated, and virtual ions are cast onto the surface. By means of ray tracing, the local environment of where the ions impact is evaluated. The sputter yield, reflection coefficient and the angular distributions of both reflected projectiles as well as sputtered ejecta are then taken from the 1D BCA simulations for the given parameters of the impact. Outgoing particles are further traced. Reflected projectiles may cause secondary sputtering if they hit another elevation on the surface. Recoils are redeposited in this case, and are counted as sputtered if they reach empty space.

Besides performance benefits due to the simplified assumptions over fully three-dimensional BCA calculations, SPRAY has the advantage that it detaches surface morphology from the calculation of collision cascades in the BCA picture. While the collisional physics is accounted for solely in the 1D input, SPRAY maps these data onto a given surface. Any changes from flat surface sputter yields are thus uniquely determined by the sample morphology.

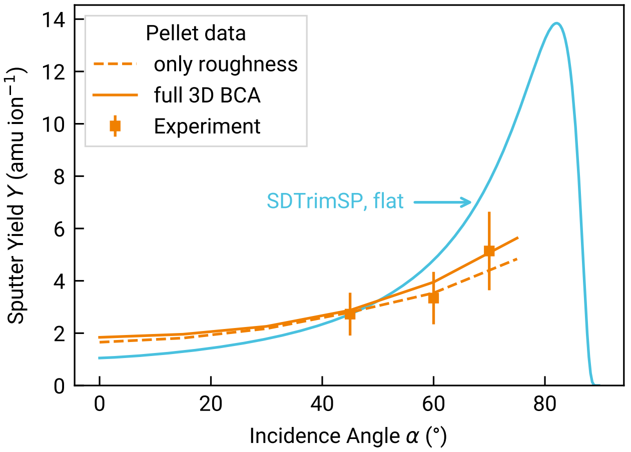

Supplementary Figure 1. A comparison of 4 keV He sputter yield data for the rough regolith pellets as given by the experiments (squares), SPRAY simulations accounting for the surface roughness (dashed line) and SDTrimSP-3D with full calculations of the collision cascades (solid orange line).

Supplementary Figure 1 shows SPRAY results (labelled "only roughness", dashed orange line) for 4 keV He irradiation of the pellet outlined in the main manuscript. They are compared to the full SDTrimSP-3D calculation ("full 3D BCA", solid orange line) and experimental data, as well as 1D Simulations by SDTrimSP given in blue and annotated in the figure. Note that all SDTrimSP based data (1D and 3D, as well as SPRAY inputs) are treated the same as in the main manuscript: parameter adaptations according to the approach by Szabo *et al.* [2] and scaled according to Fig. 1 (main manuscript) to correct for the remaining overestimation. For a broad range of incidence angles, SPRAY and SDTrimSP-3D co-

* broetzner@iap.tuwien.ac.at

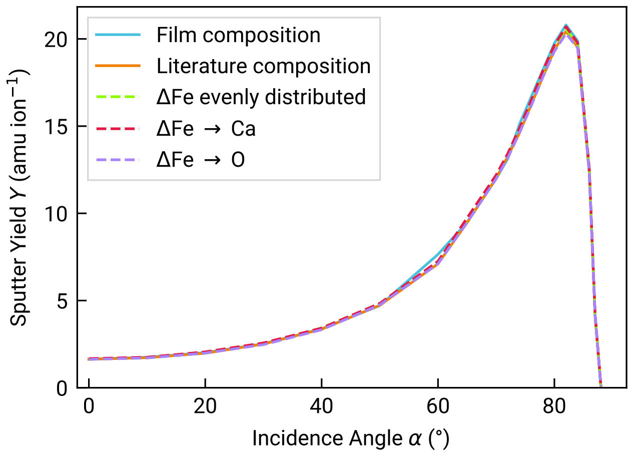

Supplementary Figure 2. Sputter yields for 4 keV He of the lunar sample with the experimentally achieved composition (blue), the literature composition (orange) as well as three variation of how to distribute the over-abundance of Fe in the film (dashed lines).

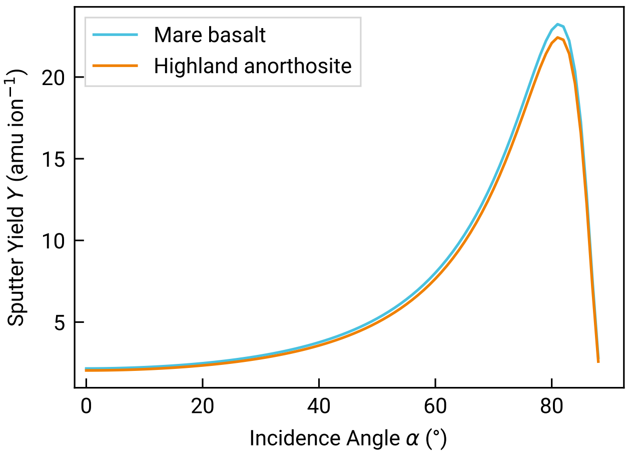

Supplementary Figure 3. Comparison of the sputter yields given by SpuBase for 4 keV He irradiation of a mare basalt sample (blue) and a highland anorthosite (orange).

incide. Only for grazing incidences, a slight difference becomes apparent. However, both curves still lie within the experimental uncertainties and neither can therefore be favoured from an experimental point of view. We can thus conclude that the influence of the surface structure is accurately accounted for in SDTrimSP-3D, under the condition that the surface input is a good representation of the physical reality and that the material-dependent flat surface sputter yields are reasonable.

Influence of Surface Composition Variations

To investigate the effect of variations in sample composition on the sputter yield, we carried out additional SDTrimSP simulations. In particular, the thin films have over-stoichiometric abundances of Ca, Fe and Ti compared to the literature values [3, 4] given in Table 1 of the main manuscript. We compare the sputter yields of the composition as achieved by the PLD process with the literature composition, and, as a particular case study, we decreased the content of Fe, the heaviest of the species, by 1.1 at.% to achieve the documented concentration for this element. Because the composition has to sum to unity, we discuss three ways to distribute the newly available 1.1 at.% of concentration:

1. Evenly distributed among all other species
2. Exclusively added to Ca, the heaviest element in the sample (besides the trace amount of Ti)
3. Exclusively added to O, the most abundant element

All additional simulations were carried out using the approach by Szabo *et al.* [2]. The results are shown in Supplementary Figure 2, and it is evident that for neither case, any significant difference is achieved. Therefore, even though the 68501 sample may not have been precisely transferred to the QCM by the PLD process, these deviations do not change our results. From a physical point of view, in the sputtering of compound samples the bulk stoichiometry governs the ratios of particle fluxes in the steady state [5]. A species of minor abundance, like Fe or Ti, will thus only contribute a small fraction of the sputtered particle fluxes, and these small variations do not influence the mass yield, even though these species may have higher atomic masses.

On a similar note, the same concept is observed in SpuBase, where the underlying simulations have been carried out with a different binding energy approach. SpuBase comes with sample compositions representative of both lunar highland anorthosite and mare basalts. The sputter yields for these are shown in Supplementary Figure 3. These two samples differ in their Ti and Fe content by roughly 3 at.% points and 0.6 at.% points, respectively. Nonetheless, the variation in total sputter yield is still significantly smaller than our experimental uncertainty and could not be resolved by the QCM outside the error bars.

In summary, even though our final thin film might geologically describe a slightly different sample, any deviations in the sputtering properties therefrom cannot be resolved experimentally. Our results are thus applicable across a wider range of lunar mineralogy rather than Apollo 16 soil 68501 alone. Significantly different sputter yields, i.e. with uncertainties that do not overlap, are only expected when exchanging elements that are both abundant and have considerable atomic masses, therefore describing different minerals altogether. This case is shown in Fig. 3 of the main manuscript.

Supplementary References

[1] C. Cupak, P. S. Szabo, H. Biber, R. Stadlmayr, C. Grave, M. Fellinger, J. Brötzner, R. A. Wilhelm, W. Möller, A. Mutzke, M. V. Moro, and F. Aumayr, Sputter yields of rough surfaces: Importance of the mean surface inclination angle from nano- to microscopic rough regimes, Applied Surface Science **570**, 151204 (2021).

[2] P. S. Szabo, H. Biber, N. Jäggi, M. Brenner, D. Weichselbaum, A. Niggas, R. Stadlmayr, D. Primetzhofer, A. Nenning, A. Mutzke, M. Sauer, J. Fleig, A. Foelske-Schmitz, K. Mezger, H. Lammer, A. Galli, P. Wurz, and F. Aumayr, Dynamic Potential Sputtering of Lunar Analog Material by Solar Wind Ions, The Astrophysical Journal **891**, 100 (2020).

[3] C. Meyer, Lunar Sample Compendium, 68501 and 68510 (2010).

[4] B. M. Bansal, S. E. Church, P. W. Gast, N. J. Hubbard, J. M. Rhodes, and H. Wiesmann, The chemical composition of soil from the Apollo 16 and Luna 20 sites, Earth and Planetary Science Letters **17**, 29 (1972).

[5] W. Eckstein, Sputtering Yields, in *Sputtering by Particle Bombardment: Experiments and Computer Calculations from Threshold to MeV Energies*, Topics in Applied Physics (Springer, Berlin, Heidelberg, 2007) pp. 33–187.
